# Human Visual Search Does Not Maximize the Post-Saccadic Probability of Identifying Targets

**DOI:** 10.1371/journal.pcbi.1002342

**Published:** 2012-02-02

**Authors:** Camille Morvan, Laurence T. Maloney

**Affiliations:** 1 Department of Psychology, New York University, New York, New York, United States of America; 2 Center for Neural Science, New York University, New York, New York, United States of America; 3 Department of Psychology, Harvard University, Cambridge, Massachusetts, United States of America; Northwestern University, United States of America

## Abstract

Researchers have conjectured that eye movements during visual search are selected to minimize the number of saccades. The optimal Bayesian eye movement strategy minimizing saccades does not simply direct the eye to whichever location is judged most likely to contain the target but makes use of the entire retina as an information gathering device during each fixation. Here we show that human observers do not minimize the expected number of saccades in planning saccades in a simple visual search task composed of three tokens. In this task, the optimal eye movement strategy varied, depending on the spacing between tokens (in the first experiment) or the size of tokens (in the second experiment), and changed abruptly once the separation or size surpassed a critical value. None of our observers changed strategy as a function of separation or size. Human performance fell far short of ideal, both qualitatively and quantitatively.

## Author Summary

Vision is most sensitive to fine detail at the center of gaze (the fovea). We typically move our eyes several times a second to build up an accurate picture of the world around us and find objects of interest. Very recently, researchers have developed models of how a visual system like ours could search a scene for a specific target with the smallest possible number of eye fixations. In two experiments, we tested the assumptions underlying such models. We set up visual “games” in which observers were rewarded for their performance in moving their eyes once to recognize simple targets. To do well (earn the maximum possible reward), observers had to move their eyes according to the predictions of recent models of eye movement. We found that our observers failed to choose optimal eye movement strategies and failed to maximize their potential winnings. Our results suggest a simpler picture of eye movement selection, driven by a few simple heuristic rules that lead to good but not optimal performance in everyday tasks.

## Introduction

For many detection and discrimination tasks, performance decreases rapidly with increasing distance from the center of vision. Observers overcome this limitation by making discrete eye movements (saccades) as often as three times per second, in effect scanning the environment. Such serial scanning is not limited to humans or to the visual modality. It is commonly found whenever the sensory range is limited spatially but the sensors can be displaced. Examples include exploratory whisking by rats [1] echo-location by bats [2], and haptic exploration by humans [3].

The pattern of eye movements depends on the observer's goals [4], [5], [6], [7]. In visual search, for example, the observer is searching for a specified target within the visual field. Following each eye movement the visual system gains access to new information as a result of the most recent eye movement and must decide whether to terminate the search because the target has been located, to continue the search by planning a further eye movement, or to abandon the search. If the search is continued then a key question is, how does the visual system plan the next saccade given the visual information gathered so far?

Models of eye movement planning fall roughly into two categories. The first class, salience models, uses the current retinal image to assign a numerical measure called salience to each location in the retina [8], [9], [10]. Salience is often linked to physical measures such as luminance or local contrast. Salience models differ in how salience is computed and in how the visual system uses the salience map to plan the next saccade.

**Figure 1:**
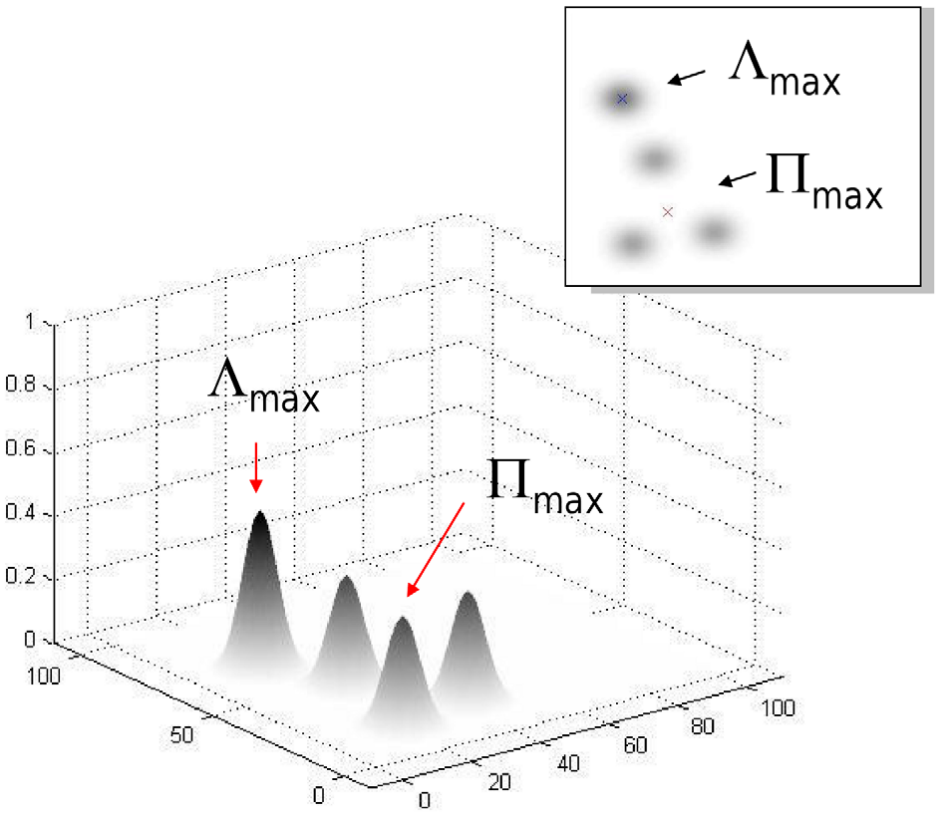
A likelihood map. The map is a plot of the likelihood that a specified target is present at each retinal location. The task is to select the retinal destination for the next saccade. Two strategies are illustrated. The first computes a saccade to the retinal location with highest likelihood (Λ_max_). The second computes a saccade that maximizes the probability of identifying the target location after the first saccade. We refer to the destination of this saccade as the Π_max_. Najemnik & Geisler (2005) emphasized that Λ_max_ and Π_max_ can be different when one takes into account the information gathering capabilities of the retina away from fovea and considers adaptive strategies that plan future saccades based on information gathered in previous saccades.

Models of the second class, optimal statistical models, are designed to optimize a specified criterion [11]. These models take into account the visual sensitivity of the eye across the retina and make use of all of the information gained in past searches to plan a sequence of saccades that, for example, minimizes the expected number of saccades needed to locate the target [12], [13], [14], [15], [16]. The information gathered during initial fixation and with each successive saccade is a measure of the likelihood that the target is at each possible retinal location, a likelihood map (Figure 1).

While the ability of such statistical models to predict eye movements behavior in natural scenes has been challenged [17], [18], [19], [20], [21], [22] and alternative models have been proposed, in particular that of Tatler and colleagues [23] incorporating high level features, statistical models allow to model ideal (optimal) behavior and compare human performance to ideal [11].

The differences between salience models and statistical models are less than they first appear to be. Likelihood, for example, is arguably a candidate measure of salience. However, a major difference between the two classes of model is the rules for planning the next saccade. With salience models these rules are typically ad hoc, chosen to capture known features of human visual search. They usually propose that the next saccade go to the currently “most salient” location but with some mechanism inhibiting return to those that have already been searched (inhibition of return [24], [25]). The planning algorithms for statistical models, on the other hand, are dictated by the requirement that search be optimal by a previously specified criterion. The modeler typically has no further choices once visual sensitivity across the retina is measured and the criterion to be optimized is selected.

Recently, Najemnik & Geisler [13] analyzed the performance that could be expected of a statistical model designed to minimize the number of saccades needed to locate a target. Given the current likelihood map, it is intuitively appealing to plan the next saccade to the location most likely to be the target, the maximum likelihood point denoted 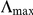 in Figure 1. Najemnik & Geisler [13] demonstrate that the correct optimal strategy, minimizing the expected number of saccades to locate a target, typically aims at a location 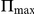 that need not coincide with 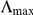. 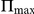 is the location that allows the visual system to best use its extra-foveal retinal sensitivity to evaluate multiple locations simultaneously as illustrated in Figure 1 and in the accompanying inset. The likelihood of a target at the location 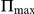 may be very low, as indicated in Figure 1. Moreover, the optimal strategy also considers possible information gathered from future searches contingent on information gained from the current, much as a strong chess player thinks beyond the immediate consequences of his current move.

Najemnik & Geisler [13] compared human performance in searching for a small Gabor patch in 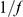 noise background to the predictions of an optimal statistical model and found qualitative agreement between the model and performance, at least in overall performance.

One difficulty in comparing performance between human and model is that the stimuli are complex and it is difficult, to predict trial by trial, where the ideal observer should fixate. Here we present a simplified visual search task which allows us to test whether the visual system uses its extra-foveal sensitivity (as Najemnik & Geisler [13] propose) to minimize the number of saccades required to identify the target.

**Figure 2:**
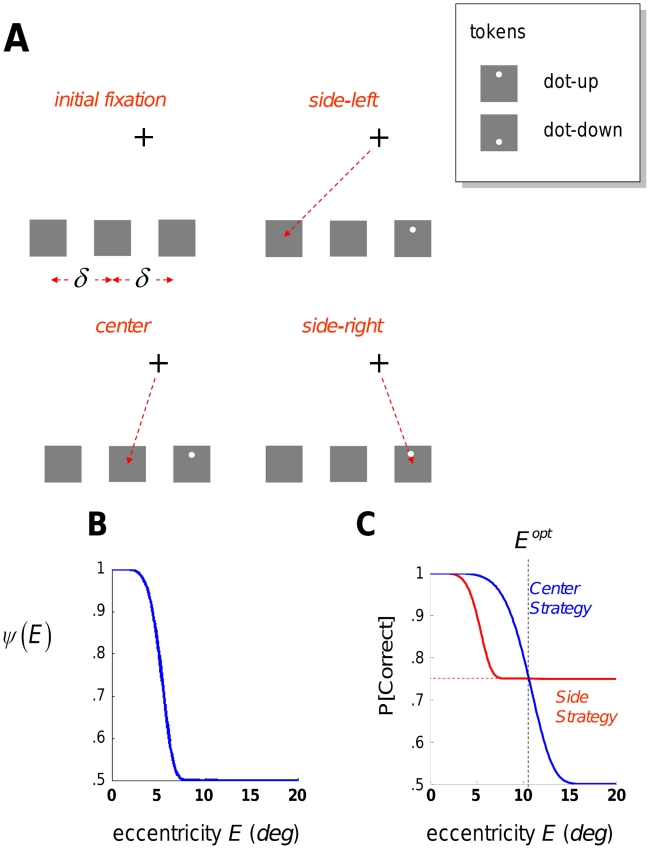
Experiment 1: The configural task and saccadic strategies. A. There were three tokens (grey squares) present in the visual search array. They are equally spaced horizontally with separation *δ* (deg). The observer initially fixated the fixation cross that is either above the central token or below (shown as above). The observer chooses one of three saccadic strategies, side-left, center, or side–right, saccading to one of the three tokens. If the observer saccaded to any other location, the trial was terminated. Once the saccade was completed, the configural target appeared with equal probability in either the left or right token but never in the center token. The target was one of two configurations shown in the inset, labeled dot-up or dot-down. In the figure the target appears on the right. The other side token and the center token remained grey squares. The observer's task was to judge whether the target is dot-up or dot-down. B. The observer's ability to correctly judge whether the target is dot-up or dot-down depended on the retinal eccentricity of the target E. It was captured by a retinal sensitivity function *ψ(E)* that varied from nearly 1 (fovea) to 0.5 (far periphery). The retinal sensitivity function for one observer (S01) is shown in the figure. The retinal sensitivity function and separation *δ* determined the choice of strategy that maximized the observer's probability of a correct response in the task. C. For observer S01, the expected probability correct corresponding to the center strategy (always saccade to the center token) is plotted in blue and the expected probability correct corresponding to the side strategy (always saccade to one of the side tokens) is plotted in red. *P[Correct|Center]* is greater than or equal to *P[Correct|Side]* from 0° to a separation *E^opt^*, the optimal switch point. Beyond the optimal switch point, *P[Correct|Side]* is greater than *P[Correct|Center]*. For separations between 0° and 3° about the difference between the two strategies is slight. However, the observer seeking to maximize expected probability correct for separations between 6° and 20° (the range of Experiment 1) should adopt the center strategy for separations less than *E^opt^* and then switch to the side strategy. The value of *E^opt^* depends on the observer's retinal sensitivity function *ψ(E)* and may differ for different observers.

In this task, the observer makes only one saccade per trial and we restrict the observer's possible choices of saccadic destinations to three. The observer must saccade to one of these three possible locations, marked by gray squares arranged horizontally, above or below his initial fixation (Figure 2A). When the observer has completed the saccade, the target appears at either the left or right location, but never in the center. The trial is aborted if the observer tries to execute a second saccade.

The target consists of a grey square and a small white dot that is either near the top of the square (dot-up configuration) or near the bottom (dot-down configuration). The two configurations are shown in an inset in Figure 2A. The observer's task is to discriminate whether the target is dot-up or dot-down. He receives a small amount of money for each correct discrimination.

The observer's probability of correct discrimination is determined by his retinal sensitivity function 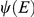 where 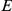 (eccentricity) is the distance from the fovea to the target. For the discrimination task we employed, 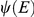 is a decreasing function of 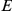. We plot an example of 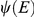 versus 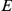 for one observer in Figure 2B.

The observer has only three possible choices of strategy. He may saccade to the leftmost token, the center token, or the rightmost. If the observer adopts the center strategy, then the separation between where the observer is fixated and the target on left or right is just the spacing between the locations (denoted 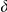 in the Figure 2A). The probability of correct discrimination is
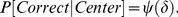
(1)


If the observer adopts a side strategy then the separation will be either 0 (if he has chosen the location where the target appears) or 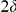 (if he has chosen the location on the side opposite to the location where the target appears). Since the target appears at the left or right location with equal probability, the observer's probability of correct discrimination is
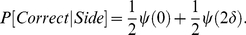
(2)


In Figure 2C we plot 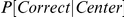 and 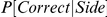 for the observer whose retinal sensitivity map is shown in Figure 2B. If the tokens are close together (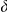 is small) then the probability correct is close to 1 for both strategies. When the separation is between 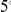 and 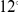, use of the center strategy would lead to higher probability correct. Beyond the point marked 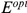 use of the side strategy would maximize expected probability correct. This critical value is determined by the observer's retinal sensitivity function. If the human observer is using his peripheral sensitivity to maximize the probability of correct discrimination, we would expect an abrupt change in strategy when the separation of center and side tokens exceeds 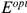.

In Experiment 1 we first measured observers' retinal sensitivity functions. In the main part of the experiment, observers chose between center and side strategies as we varied separation 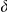 over the range 8 to 24 degrees. Observers received a small monetary reward for each correct discrimination. We compared observers' choices of strategy (center or side) to the choice of strategy maximizing expected gain. The observer maximizing expected gain would pick the strategy, center or side, offering the larger probability correct in Equations 1 and 2, switching strategy at the optimal switch point 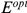.

In Experiment 2 we varied the size of the targets rather than distance to manipulate 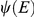. Observers chose between the same array of tokens in Figure 2A but now the tokens varied in size. There is still an optimal point in 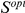 where the observer should switch from a center strategy to a side strategy but now it is expressed in size.

**Figure 3:**
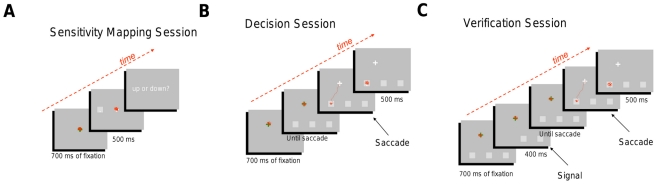
Experiment 1: A. Retinal mapping session. The observer first fixated a fixation cross for 700 msec. Then a target appeared on the right or left of the fixation point. The target was displayed for 500 ms and then disappeared. A response screen was displayed until observers indicated their response. B. Decision session. The observer first fixated a fixation cross for 700 msec. Then square markers appeared at the three locations to which the observer was permitted to saccade. Once the observer completed the saccade, the target configuration appeared at one of the side locations chosen at random. The observer then judged whether the target was dot-up or dot-down. We compared human performance to performance maximizing probability of correct discrimination. C. Verification session. The protocol was the same as the Decision session except for the addition of a 400 ms phase, before the saccade, where one of the 3 tokens disappeared. This token indicated the position that the observer should saccade to. If the observers made his saccade to another location (fixation position not within a 1° radius of the indicated object) the trial was aborted and replayed later. Actual eye movement data for one trial for one observer is superimposed in red on the stimulus arrays.

Each experiment consisted of three phases, sensitivity mapping, decision, and verification, illustrated in Figure 3 and described in the Methods section. In the sensitivity mapping phase, we measured sensitivity for the visual task for different eccentricities of targets (Experiment 1) and for different sizes of targets at different eccentricities (Experiment 2). In the second (decision) phase, we tested human ability to select eye movements that maximize expected gain. In the last phase (verification), we repeated the decision phase but forced the observer to make the saccade that our model (see Methods section) predicted would maximize expected gain. By doing so, we verified that, had they followed this strategy, they would have increased their expected gain to the maximum possible expected gain predicted by the model.

In the separation and size experiments, we assumed that the target is always presented and we considered only the first saccade. If we were to modify the task slightly so that, although the grey squares appeared, the target configuration (dot-up or dot-down) was not presented on one half of the trials (that is, all of the grey squares were uniform, without a marked configuration), then on trials where the observer fails to detect the location of the configuration after one saccade, he must make one or two additional saccades to determine if the target configuration is present at all and in what configuration. The strategy in our task which maximizes probability correct also minimizes the number of saccades needed to be correct in this modified task.

In the experiments reported, we interleaved separations (Experiment 1) and sizes (Experiment 2) rather than presenting the same separation or size repeatedly in in a single experimental block. Observers could potentially learn the blocked task by simply trying different saccadic strategies and seeing which is more rewarding as a function of separation (Experiment 1) or size (Experiment 2). In effect they learn to pair strategy and separation/size by reinforcement learning. But the prediction of the class of statistical models we consider is that observers will take into account their own retinal sensitivity in planning saccades without an extensive history of reinforcement [26]. Reinforcement learning plays no role in these theories.

## Results

### Sensitivity mapping phase

**Figure 4:**
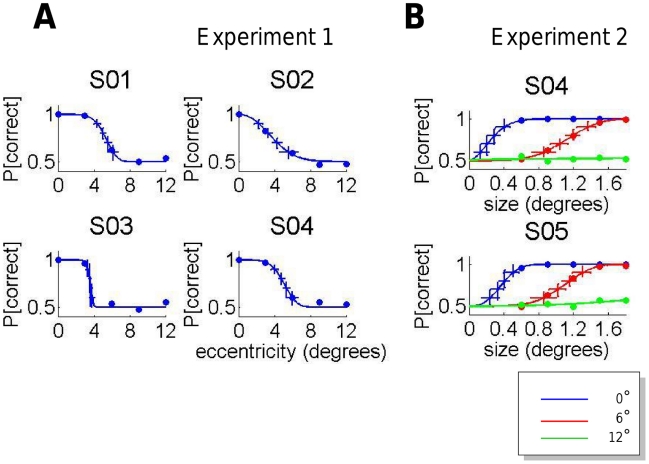
Retinal sensitivity mapping. A. Experiment 1. The observer's probability of correctly identify the stimulus configuration (dot-up or dot-down) is plotted as a function of the eccentricity at which the configuration was presented. Data is presented for all five observers. The smooth curve is a maximum likelihood fit of a psychometric function. The observer's probability of correctly identify the stimulus configuration (dot-up or dot-down) is plotted as a function of the stimulus size for stimuli presented at each of three retinal eccentricities. B. Experiment 2. Data is presented for both observers. Observer S04 in Experiment 2 was the same observer as Observer S04 in Experiment 1. See text.

The sensitivity mapping plots are presented in Figure 4A for Experiment 1 and in Figure 4B for Experiment 2. The maps show the percentage of correct responses as a function of Eccentricity for Experiment 1 and Size for Experiment 2. The results for each observer are used to predict the ideal observer performance in the decision phase.

### Decision phase

#### Saccadic choices

We analyzed the experimental data by first identifying observers' decisions. For each trial, the saccade was categorized as being directed to the closest token, computed in Euclidean distance (see Figure S1 in Text S1 and discussion).

#### Strategy

**Figure 5:**
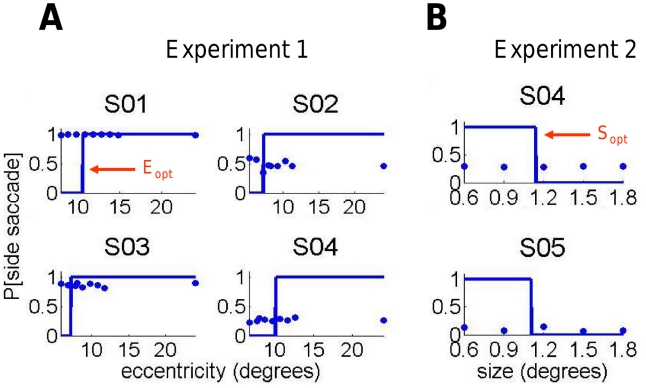
Strategy choice. A. Experiment 1. For each observer we plot the proportion of time they picked either the side strategy as a function of eccentricity of the side locations markers. The proportions predicted by an ideal observer maximizing probability of correct response are shown as solid blue curves. These are step functions, going from 0 to 1 at the optimal switch point for the observer which is computed from each observer's retinal sensitivity function. Observers failed to shift strategy with changes in eccentricity. B. Experiment 2. For each observer we plot the proportion of time they picked either side strategy as a function of size of the targets.

Figure 5 shows the probability to saccade to a side object as a function of the tokens separation for Experiment 1 (Figure 5A) or size for Experiment 2 (Figure 5B). For each observer and experiment, we obtained 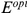 and 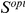, the optimal switch points, using the psychometric function estimated from the mapping data. The optimal strategy is plotted as a solid line. The optimal strategies for our choices of 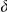 are to saccade to the middle for short separations or big sizes, and vice versa for the big separations and the small sizes. The optimal strategy switches (vertical line) at a specific value 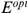 or 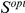
of the independent variable from a 0% probability of a side saccade (i.e. 100% probability of a center saccade) to a 100% probability of a side saccade (either left or right).

If observers were optimal their probability of choosing a side strategy would follow this step function, and any deviation from this step function reduced their expected gain and probability correct. The results, shown as blue dots (Figure 5) are unequivocal: observers were markedly suboptimal. Not only did they fail to switch strategy at 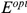 or 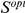, but more strikingly, they did not change their strategy at all as a function of the separation or size of the tokens.

#### Saccadic latencies

Some observers took their decisions very quickly while others were slow. Also, within a given observer there was some variability in the speed of their decisions. We examined whether the speed with which observers made their decision correlated with how “good” the decision was by plotting the likelihood of the saccades to be correct as a function of their latency. As shown in Figure S2 in Text S1, the quickness of the decision does not seem to affect whether the decision was closer to or further from optimal or not.

#### Saccade length

Recall that the distances from initial fixation to the two side objects were not the same. Whenever the observer chose to saccade to one of the side objects, they preferred the nearer. The proportion of side strategy saccades to the nearer of the two objects were 0.85, 0.64, 0.95, and 0.86 for the four observers in Experiment 1, 0.99 and 0.77 for the two observers in Experiment 2.

#### Inter-trial dependencies

One possible explanation of observers' sub-optimal performance is that switching strategies (side to center or vice versa) might entail a “cognitive cost”. We tested whether observers tended to repeat strategies by computing the conditional frequency with which a choice of a given strategy on one trial is followed by a choice of the same strategy on the following across all trials. We compared this conditional frequency to the (non-conditional) frequency with which observers used a given strategy estimated across all trials. The conditional frequency of using a specific strategy was significantly greater than the non-conditional frequency: 0.76 versus 0.5 for the side strategy and 0.6 versus 0.5 for the center strategy. One-tailed t-tests indicated that the difference is statistically significant in both cases with p = 0.04 and p = 0.03 respectively for side and center strategies. We conclude that some observers tended to repeat strategies above chance.

The observed inter-trial dependencies provide some characterization of human performance in our task beyond the simple claim that it is sub-optimal. Of course, given that the trials were randomized, any evidence of inter-trial (sequential) dependencies in choice of saccadic strategy is further evidence for sub-optimal choice of strategy. We cannot conclude that these dependencies account for failures in human judgment in our task since any causal connection may well be in the reverse direction: the observer, ignorant of the correct strategy, may tend to default to whatever he chose on recent trials.

Similarly, the experimental conditions (separation or size) were interleaved and randomized in both experiments. On occasion, a particular separation or size was followed by the same separation or size. We considered the possibility that observers chose the optimal strategy for the repeated condition with greater frequency. It was not the case: the probability to pick an optimal saccade was 0.57 on average, and 0.59 for repeated conditions, the difference was non-statistically significant on one-tailed t-test (p = 0.19).

#### Performance (Gain)

**Figure 6:**
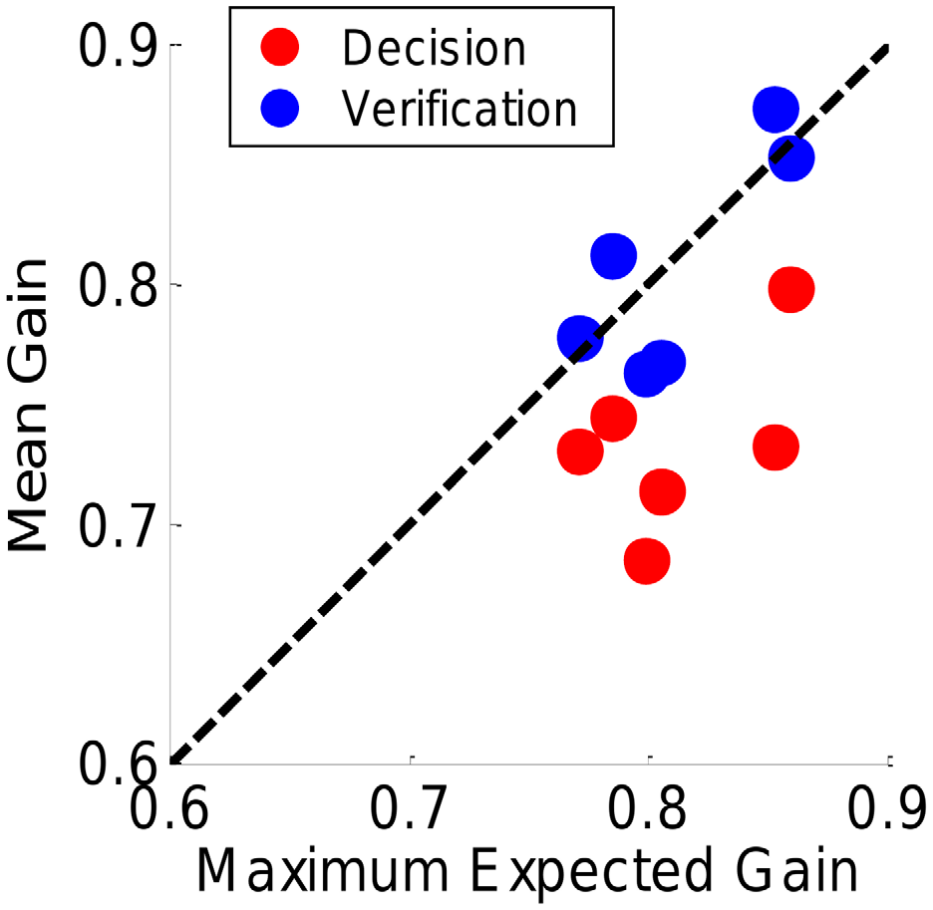
Comparison to Maximum Expected Gain. Each observer's mean gain in the decision phase (in red) and in the verification phase (in blue) is plotted versus the maximum expected gain possible for that observer. The red points are consistently below the 45 degree line indicating that observers failed to maximize expected gain as predicted by the model in the decision phase. The blue points on the other hand are scattered around the 45 degree line. Observer's gain approached the maximum possible gain when the observer executes the eye movement strategy that maximizes expected gain as predicted by the model.

In the verification phase we directly confirmed that observers could have increased their gain by employing the optimal strategies predicted by our model. The trials in the verification phase were identical to those in the decision phase excepted that on each trial the observer was instructed to saccade to the location that we predicted would maximize his expected gain (see Methods). We plot mean gain for each observer in the decision phase (red circles) and in the verification phase (blue circles) in Figure 6. In the decision phase, observers did not maximize expected gain and their choices of strategies reduced their expected winnings by 9% on average (max = 17%, min = 6.1%, SD = 0.0398). When forced to choose optimal strategies on each trial, observers' gain increased and is indistinguishable from maximum expected gain as predicted by the model.

In the analyses just reported here, we used estimates of retinal sensitivity function 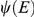 from the Sensitivity Session to predict the visual strategies that would maximize expected gain in the Decision Session. However, in the Decision Session, each visual judgment was preceded by a saccade while in the Sensitivity Session it was not. There is considerable evidence that a preceding saccade can briefly alter perception [27], [28], [29] and we can conjecture that it may also alter sensitivity.

However, we can readily estimate 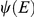 based on data in any of the three sessions and use these estimates to predict the visual strategy maximizing expected gain in the Decision Session. In the Figure S3 of the Text S1 we compare observers' data to these predictions. Inspection of this plot shows that each observer's data is inconsistent with the optimal strategy based on 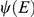 in any session. Whichever session we choose to estimate 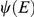, we reach the same conclusion: observers did not adapt their strategies to the separation of the tokens (Experiment 1) or to changes in size (Experiment 2).

To test whether observers maximized expected gain, we performed a nested-hypothesis test [30] comparing each observer's winning to those of an observer maximizing expected gain. The results for Experiment 1 with all observers combined indicated that observers overall did not maximize expected gain (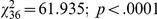) and that three of the four observers considered in isolation did not maximize expected gain: S01 (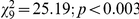), S03 (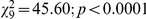) and S04 (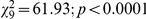). The winnings of the remaining observer S02 were not significantly different from maximum expected gain (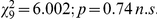). For Experiment 2, the overall test lead to rejection of the hypothesis that the observers maximized expected gain (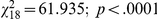) and to rejection of the hypotheses that each observer maximized expected gain (S04: 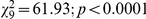, S05: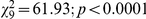).

## Discussion

### Summary

We reported two experiments intended to determine whether observers correctly employed their extrafoveal retinal sensitivity to optimize visual search. Each experiment consisted of three phases, sensitivity mapping, decision, and verification. In the sensitivity mapping phase, we measured each observer's retinal sensitivity as a function of target eccentricity (Experiment 1) and/or target size (Experiment 2).

On each trial of the decision phase, observers first executed a saccade to one of three retinal locations, left, center or right (Figure 2A). Following the saccade, a target would appear at either the left or right location but never in the center location. The observers then attempted to discriminate whether a small white dot within the target was near the top or bottom of the target.

Their probability of success in discriminating depended on the location to which they had saccaded and the location at which the target appeared. Observers received a monetary reward for each correct discrimination and their challenge was to decide which location to saccade to so as to maximize their expected gain. We refer to their choices as a saccadic strategy. There were only three possible strategies, left, center, or right, and two of these strategies, left and right, were effectively equivalent (see Methods). We refer to them collectively as the side strategy.

A center strategy led to better discrimination for smaller eccentricities (Experiment 1) or larger sizes (Experiment 2). Whether the target appeared on the left or right side, the small eccentricity (large size) meant that the observer could discriminate above chance while fixated at the center location. For large enough eccentricities in Experiment 1, the center strategy resulted in performance near chance. In contrast, either side strategy resulted in better performance since, if the target appeared on the same side as the observer chose to saccade to, then he could readily discriminate it. This would occur on half the trials and on the remaining trials, when the target appeared on the side not chosen, the observer would be near chance in responding. Overall, the side strategy would lead to performance better than that expected with the center strategy. See Figure 2C. The same conclusion holds in Experiment 2 where we varied size.

Consequently, as the experimenter increased the eccentricity of the side locations or decreased the size of the target, the observer optimizing expected gain or, equivalently, probability correct, should switch from a center strategy to either one of the side strategies at a specific optimal switch point.

We used the data from the sensitivity mapping phase to predict the optimal switch point as a function of eccentricity or in size for each observer. We compared observers' choices of strategy to the choices predicted to maximize their probability correct in the discrimination task.

None of our 6 observers switched strategy at the optimal point. All had evident, idiosyncratic biases, toward either the side or center strategies, but, most strikingly, they chose the center and side strategies equally often for all eccentricities and sizes of target. They did not adapt their strategy to the stimulus configuration at all.

In a separate verification phase we reran the main part of the experiment but now indicating to the observer where to saccade on each trial, “forcing” the observer to adopt the saccadic strategy that our model predicted would maximize probability correct and expected gain. We found that observers' mean gain increased when they executed the strategy predicted to maximize expected gain and that their mean gain was in good agreement with the maximum expected gain predicted by the model.

In summary, observers did not respond to variations in token separation and size at all. They apparently ignored the independent variable in each of the two experiments. We had expected that, for example, their performance might be qualitatively consistent with that of the ideal observer. That is, they might abruptly switch strategies at some separation (size) but not at the separation (size) that maximized expected gain. Or they might have been inconsistent in choosing strategies but only near the switch point so that probability of picking the side strategy might smoothly decrease from 1 to 0 instead of following the ideal step function. Neither of these outcomes occurred. We see no evidence that the visual system is sensitive to the factors we varied in the experiment.

Humans sometimes do not make single saccades even when it is possible instead producing two or three saccades [31]. We considered the possibility that this particular aspect of our protocol is responsible for observed sub-optimality. An argument against this possibility is the ease with which our subjects adapted to the task. The ratio of excluded trials due to blinks or second saccade combined was between 8% and 24% of the trials (15% on average).

If the observer's saccade did not arrive within one degree of a token, we terminated the trial. This occurred on between 2.4% and 34% of trials (mean 17%), across observers. However, the horizontal and vertical standard deviations of saccades in our experiment (see Figure S1 in the Text S1) are large compared to the one degree cutoff we imposed and, even if the observer attempted to saccade to the center of a token on every trial, many of the resulting saccades would fall outside the one degree limit. Hence, we cannot infer that a failure to saccade to within one degree of a token (the criterion for success) indicates that the observer intended to saccade anywhere other than to the token. In particular, there is no basis to conclude that normal eye movement planning and execution has been altered by the constraints we impose.

Had we used a less stringent criterion for termination of a trial (or imposed no criterion), observers might have chosen to saccade to a location away from any of the three tokens. Our analysis depends on knowing what strategies are available to the observer and which of these they chose. We also verified that the observer's distributions of saccades to each token were approximately centered on the token and not off to one side. The distributions of saccade endpoints for all observers are show in Figure S1 of the Text S1 as well as descriptive statistics for all the observers.

If planning consumes cognitive resources then the choice of optimal plan should reflect the “cost” of these resources to the organism [32]. The key problem, though, is to develop experimental methods that allow us to demonstrate that these hidden costs are real and that they explain the observer's behavior.

In conclusion, we find little evidence that observers correctly use their visual sensitivity outside the fovea to optimize visual search.

### Heuristic based planning

Our results are in apparent conflict with the predictions of optimal statistical models discussed in the introduction [12], [13], [14], [15], [16]. Najemnik & Geisler [13], for example, asked observers to locate a Gabor patch in a 1/f field of noise. They compared human performance to ideal performance minimizing the expected number of saccades to find the target. As we explained in the introduction, the strategy that maximizes expected gain and probability correct in our task also would minimize the number of saccades needed to correctly discriminate the target configuration.

Our task is designed so that the visual system must have access to estimates of retinal sensitivity as a function of size or eccentricity in order to plan saccades that maximize expected gain. We, in effect, compared choice of saccade on each trial to the choice of saccade that would maximize expected gain, something we could do because of the simplicity of our design.

The key predictions of Najemnik & Geisler's model are more difficult to match to human performance in their experiments. They, for example, predict the length of the first saccade and find that the distributions of lengths of first saccades are matched to that of the ideal. However, this does not imply that any particular saccade, triggered by a particular combination of signal and noise, is in itself optimal or even close.

An alternative explanation for the results of Najemnik & Geisler [13] is that human visual search is based on simple heuristics analogous to those postulated in salience models. Tatler & Vincent [33] for example, presented compelling evidence that saccade selection could be better predicted by oculo-motor preferences than by visual information or task (although they did not provide evidence of predictive power of these biases relative to chance) Under this account, the visual system has heuristic preferences for saccades of certain lengths or possibly a tendency to saccade to the center of mass of clusters of objects in the periphery [34], [35]. The second heuristic, under specific circumstances, might mimic selection of the optimal point 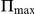 in Figure 1 not because it is the saccade that minimizes the expected number of saccades but because it lies near the centroid of a cluster of items in the visual field.

Such a heuristic-based approach may approximate ideal performance in some tasks while failing utterly in others. The experimenter who considers performance in a limited range of scenes may record behavior that approximates optimal but is in fact no more than a lucky coincidence of a heuristic rule and experimental conditions. Such “apparent optimality” is not rare in behavioral studies of animals [36] or humans [37]. And, since the stimuli of Najemnik & Geisler [13] were chosen to mimic the statistical properties of natural scenes, it is not surprising that application of visual heuristics lead to good performance in such scenes.

If human saccade decisions are based on such heuristics rather than on a computation that requires knowledge of visual sensitivity maps, we would expect a failure of adjustment when one's visual sensitivity map is changed. In fact, when observers' foveae were artificially shifted with gaze-contingent techniques, their performances in visual search were significantly worse than predicted by the ideal-observer model [38].

In contrast, we designed our simple task so that the visual system can only succeed if it has access to estimates of visual sensitivity for the range of sizes and eccentricities we considered. We compared human performance to optimal on a trial by trial basis. We conjecture that observers failed in our task because it is not well matched to the collection of visual heuristics that guide saccadic selection.

## Methods

### Apparatus

Experiments were programmed in C++ using Microsoft DirectX APIs on a Pentium 3 computer running Windows XP. Stimuli were displayed on a 19-inch Sony Trinitron Multiscan G500 monitor run at a frame rate of 100 Hz with 1280×1024 resolution in pixels. A forehead bar and chinrest were used to help the observer maintain a viewing distance of 57 cm. At that distance, the full display subtended 40.4°×30.3°. The observer viewed the display binocularly. Eye movements were recorded using an Eye Link II (SR Research, Toronto, Canada) sampling eye position at 500 Hz.

### Subjects

The subjects were NYU undergraduate students. Four subjects participated in the Experiment 1 (3 female) and two in Experiment 2 (1 female). They were unaware of the purpose of the experiment and all had normal or corrected-to-normal vision.

### Stimuli and task

Stimuli were presented against a uniform gray background (50% white). The target configuration, represented in the inset in Figure 2, consisted of a light gray square with a superimposed light gray dot at either the top (dot-up configuration) or at the bottom (dot-down configuration). The tokens subtended 1° of visual angle in Experiment 1 and between 0.6° to 1.8° of visual angle in Experiment 2. The observer's task was to report whether the target was dot-up or dot-down. Observers responded by rotating the mouse wheel in one direction corresponding to dot-up, the other corresponding to dot-down. Observers were rewarded for correct responses and they were aware that they would be rewarded. Observers were instructed to reply as accurately as possible and no time was imposed on their response. They were not given any feedback regarding their response. The maximum reward was $20.

### Experimental design

Each experiment comprised three phases, sensitivity mapping, decision and verification. We ran two experiments, in Experiment 1 we varied only the separation and in Experiment 2, only the size. The different separations in Experiment 1 were randomly interleaved, the different sizes in Experiment 2 were also randomly interleaved.

#### Sensitivity mapping phase (Figure 3A)

In the sensitivity mapping phase of each experiment a red fixation cross was first displayed at the center of the screen. After the observer's fixation was stable (eye velocity less than 10°/s and eye position within a 1° radius circle around the fixation cross) for 700 ms the cross turned white and the target appeared unpredictably to the left or right of the cross at any of five target eccentricities ranged from 0° to 12° by steps of 3°, in Experiment 1, and from 0° to 12° by steps of 6° in Experiment 2. After 500 ms, the stimulus was replaced by a response screen. The observer responded whether the target was dot-up or dot-down and had unlimited time to do so. Observer's fixation had to be stable during the entire trial or the trial was discarded otherwise. Also, blinking was not allowed. The target subtended 1° of visual angle in Experiment 1 and from 0.6° to 1.8° by steps of 0.3° in Experiment 2. Experiment 1 had 20 ( = 5×2×2) conditions repeated 20 times each (400 trials total). In Experiment 2 there were 60 ( = 3×2×2×5) conditions each repeated 25 times (1500 trials total).

#### Decision phase (Figure 3B)

In the decision phase the observers started by fixating a red cross, positioned vertically ±4° relative to the middle of the screen. The horizontal position of the cross relative to the tokens was observer-specific in Experiment 1 (see details below) and ±3° in Experiment 2. After 700 ms of stable fixation, we displayed three aligned tokens 4° above or below the fixation plane. The tokens remained visible until the end of the trial. The central token was always centered horizontally on the screen and the side tokens were equidistant on either side.

As mentioned above the fixation cross was not centered horizontally but slightly offset to the right or to the left. If it were centered horizontally it would be equidistant to each side tokens (the potential targets) but closer to the center token. But in fact, we are not testing the choice between the right and left targets but between either target or the center token. Therefore the fixation cross was displayed in a point that was equidistant to the center token and either side token. Even if the observer preferred shorter saccades, he would always have a choice between a side saccade and a central that were of equal length. In addition, given that the separations between the side token were observer specific, so was the position of the cross.

Observers were instructed to make one and only one saccade towards one of the tokens. Once the saccade landed and the eyes were stable for 50 ms, a dot appeared in one of the side tokens. The display remained visible for 500 ms if the observer's fixation remained stable and if the eyes remained within 1° of the position where the eyes landed. If the observer blinked or tried to make another saccade the trial would be aborted and replayed later in the experiment.

We chose different separations for different observers based on the observers' sensitivity mapping data. We first fit the data by a least square criterion using a four parameter psychometric function based on the logistic cumulative distribution function [39]:
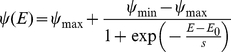
(3)where 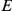 is used to denote the eccentricity of the stimulus in degrees, the value 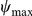 is the probability of correct classification at the fovea (typically close to 1 for our stimuli) and 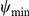 is the asymptotic probability of correct classification for large eccentricities, typically close to 0.5 which is chance performance. The parameter 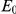 is the point where the probability of correct detection is 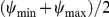. The resulting fits are shown in Figure 4. The values of 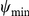 and 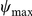 were typically close to 0.5 and 1 for all observers with 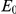 corresponding to the 75% correct point. The parameter 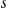 controls the slope of the psychometric function as 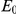. The estimated values of 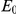 for the four observers were S01: 5.93; S02: 4.16; S03: 4.38 and S04: 4.83 degrees with mean 4.82 degrees. The separations between the side tokens for each observer included the seven values
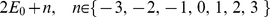
(4)and the additional values [8°, 24°]. There were therefore nine separations in total, seven chosen based on the observers' sensitivity mapping data and two common to all observers. For S01 these were, for example, [8°, 8.85°, 9.85°, 10.85°, 11.85°, 12.85°, 13.85°, 14.85°, 24°]. There were 96 trials per separation which summed to 864 trials per observer in Experiment 1. The trials were performed in 4 different sessions preceded by training trials (25 for the first session and 5 thereafter) that were not included in the analysis.

In Experiment 2 the separation between the side tokens was a constant 12°. The token sizes ranged from [0.6°, 0.9°, 1.2°, 1.5°, 1.8°] with 112 trials per size. Thus there were 560 trials performed in 7 separate sessions preceded by training trials, as in Experiment 1.

#### Verification phase (Figure 3C)

The purpose of this phase was to verify that our predictions of performance in the decision phase, based on measurements in the sensitivity mapping phase, were accurate. Instead of allowing the observer free choice of saccade locations, we instructed them which location to saccade to. Here we will show data for the cases where they were instructed to saccade to the optimal location. The design, represented in Figure 3B, was similar to the decision phase except that at the start of each trial the observer was instructed which of the three locations to saccade to: after 700 ms of initial stable fixation, one token disappeared for 400 ms and the observer could start his saccade to this indicated token as soon as it reappeared. Once the observer completed the saccade, we verified that the observer's fixation was within 1° of the indicated token.

### Model

As explained in the Introduction, there is a given separation (Experiment 1) or size (Experiment 2) at which observers should switch strategy. We call the optimal switch point 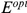 and, in Experiment 1, it is defined as the separation between the side tokens for which
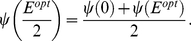
(5)


The right hand side of the equation describes the observer's performance when he has chosen to saccade to one of the side tokens. On half the trials, the target will appear at that side location and he will discriminate correctly with probability 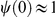 (he is fixating the target). On the other half the trials, the target will appear on the other side, a distance 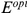 from fixation. He will discriminate correctly with probability 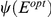. The overall probability of correct discrimination is the right hand side of Equation 3. The left hand side is the performance expected with a center strategy. Whether the target appears on left or right, it is a distance 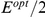 from fixation and the observer discriminates correctly with probability 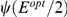. The switch point is the point at which the two strategies lead to equal discrimination performance. For eccentricities 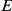 with 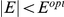, saccading to the center square results in a higher probability of correct classification. For 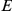 with 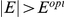, saccading to either of the side tokens leads to better performance.

We derived a similar equation for Experiment 2 but now in terms of target size. The optimal switch point 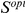 is defined by
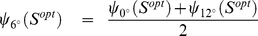
(6)with 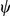 denoting the sensitivity mapping function for each observer and 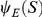
denotes the sensitivity function as a function of size for eccentricity 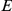. At this point, both strategies have the same probability of success. The ideal observer that maximizes expected probability correct will switch strategy precisely at 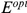 and 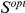.

We estimated 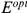 and 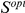 for each observer in each experiment using Equations 5 and 6 and numerical optimization. We also verified that the optimal point is unique. The 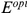 and 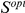 for each observer are shown together with the results in the next section.

The sensitivity functions 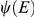 and 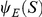 could also be estimated using the data from the decision or verification phase. We used the sensitivity function derived from the sensitivity mapping phase in the analysis reported in the main text. Using the data from either of the other two phases only led to small changes in estimated optimal switch point that do not affect our conclusions. We report those in Figure S3 in the Text S1.

## Supporting Information

Text S1 **Supplementary figures.**
(DOC)
