# Supplementary material for: Human Visual Search Does Not Maximize the Post-Saccadic Probability of Identifying Targets
Source: PLoS Comput Biol. 2012 Feb 2;8(2):e1002342. doi: 10.1371/journal.pcbi.1002342 (PMC3271024; doi:10.1371/journal.pcbi.1002342)
Supplement: Text S1 — Supplementary figures. (DOC) [file pcbi.1002342.s001.doc]

Supplementary Material: Human visual search does not maximize the post-saccadic probability of identifying targets. Camille Morvan and Laurence T. Maloney.

**Figure S1: Saccadic endpoints.** Distribution of the landing positions of the saccades for one representative observer (S04), for each separation in Experiment 1. The starting position of the eye was always along the line that crosses the screen’s horizontal center. Our experiment requires us to categorize the whether observers’ eye movements were directed to the right, left or middle token. Figure S01 shows that after data filtering (see main article) the categorization of the saccade was unambiguous, the red ellipses indicate the 1° radius selection circle area (note that the horizontal and vertical scales of the plots are not always the same and that, as a consequence, some of the circular selection areas appear elliptical in the figure).

**Table S1: Saccadic endpoints: Summary.** The mean unsigned distance (degrees of visual angle) between saccades and the center of the nearest target in each condition of both experiments. The standard deviation of the distances are shown immediately below the corresponding mean.

**Figure S2: Latencies.** Some observers planned their saccades rapidly while others spent more time in planning. We examined whether the speed with which observers planned their saccade was correlated with their probability of correct discrimination, plotting the probability of a correct choice of saccade as a function of latency. Figure S02 reveals no clear pattern between the mean latency of response and the speed of the decision across observers.

**Figure S3: Strategy choice. A. Experiment 1.** For each observer we plot the proportion of time they picked either the side strategy as a function of eccentricity of the side locations markers. The proportions predicted by an ideal observer maximizing probability of correct response are shown as solid blue curves. They are step functions, going from 0 to 1 at the optimal switch point for the observer. These predictions are based on each observer’s retinal sensitivity function derived from the Mapping Phase (in blue), the Decision Phase (in red) or the Verification Phase (in black). Observers failed to shift strategy with changes in eccentricity. **B. Experiment 2.** For each observer we plot the proportion of time they picked either side strategy as a function of size of the targets.

--------------------------------------------------------------------------------------------------------------------

**
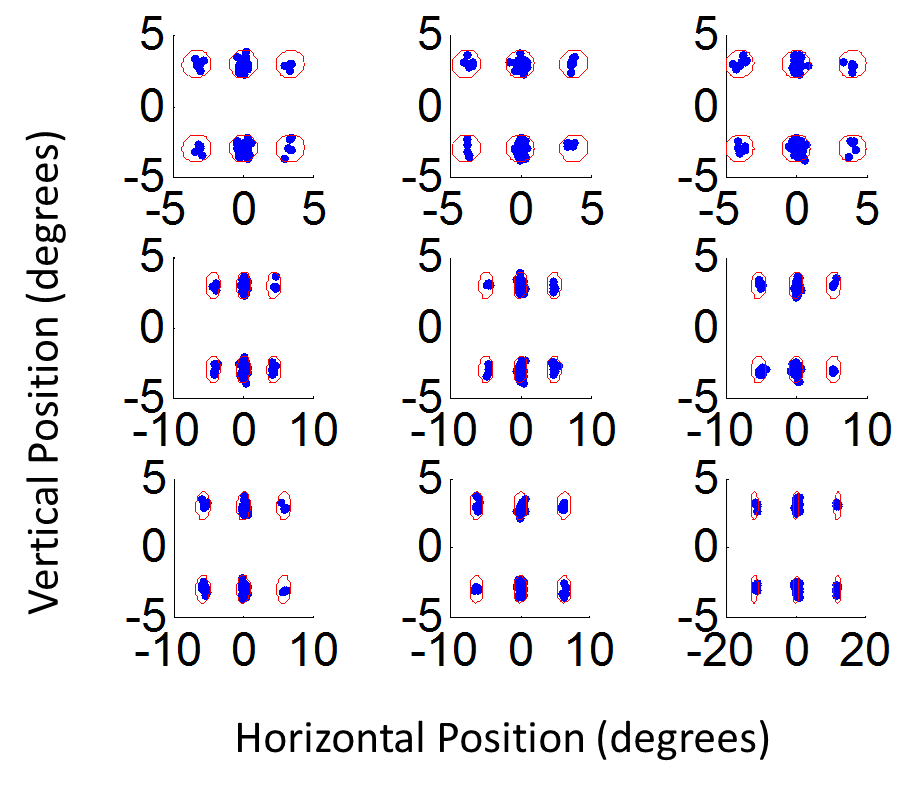
**

**Figure S1:** **Saccadic endpoints.**

| *SEPARATIONS* | | *1* | *2* | *3* | *4* | *5* | *6* | *7* | *8* | *9* |
| --- | --- | --- | --- | --- | --- | --- | --- | --- | --- | --- |
| *S1* | mean | 0.49 | 0.49 | 0.51 | 0.48 | 0.45 | 0.52 | 0.49 | 0.49 | 0.60 |
|  | sd | 0.24 | 0.22 | 0.23 | 0.23 | 0.23 | 0.23 | 0.24 | 0.25 | 0.25 |
|  |  |  |  |  |  |  |  |  |  |  |
| *S2* | mean | 0.55 | 0.50 | 0.54 | 0.61 | 0.57 | 0.53 | 0.54 | 0.57 | 0.55 |
|  | sd | 0.24 | 0.29 | 0.25 | 0.25 | 0.24 | 0.24 | 0.22 | 0.25 | 0.23 |
|  |  |  |  |  |  |  |  |  |  |  |
| *S3* | mean | 0.45 | 0.41 | 0.38 | 0.43 | 0.43 | 0.45 | 0.42 | 0.43 | 0.54 |
|  | sd | 0.21 | 0.22 | 0.21 | 0.22 | 0.21 | 0.23 | 0.22 | 0.22 | 0.22 |
|  |  |  |  |  |  |  |  |  |  |  |
| *S4* | mean | 0.35 | 0.39 | 0.40 | 0.38 | 0.36 | 0.35 | 0.34 | 0.36 | 0.46 |
|  | sd | 0.23 | 0.17 | 0.20 | 0.20 | 0.20 | 0.21 | 0.17 | 0.19 | 0.20 |
|  |  |  |  |  |  |  |  |  |  |  |
|  |  |  |  |  |  |  |  |  |  |  |
| *SIZE* |  | *1* | *2* | *3* | *4* | *5* |  |  |  |  |
| *S4* | mean | 0.55 | 0.54 | 0.55 | 0.56 | 0.60 |  |  |  |  |
|  | sd | 0.24 | 0.25 | 0.25 | 0.28 | 0.26 |  |  |  |  |
|  |  |  |  |  |  |  |  |  |  |  |
| *S5* | mean | 0.39 | 0.41 | 0.38 | 0.43 | 0.44 |  |  |  |  |
|  | sd | 0.21 | 0.22 | 0.21 | 0.25 | 0.23 |  |  |  |  |

**Table S1:** **Saccadic endpoints: Summary.**


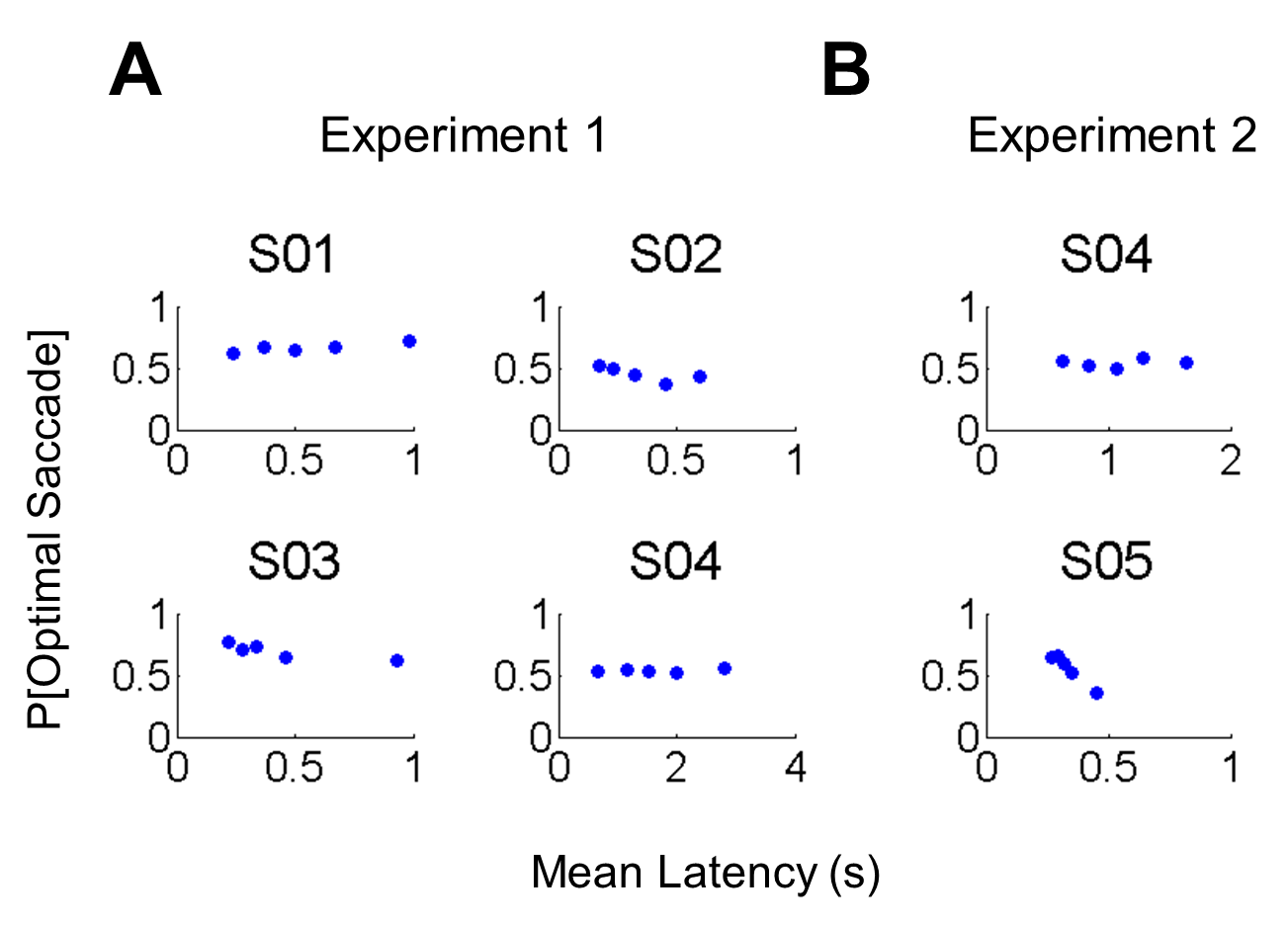


.

**Figure S2: Latencies.**

**
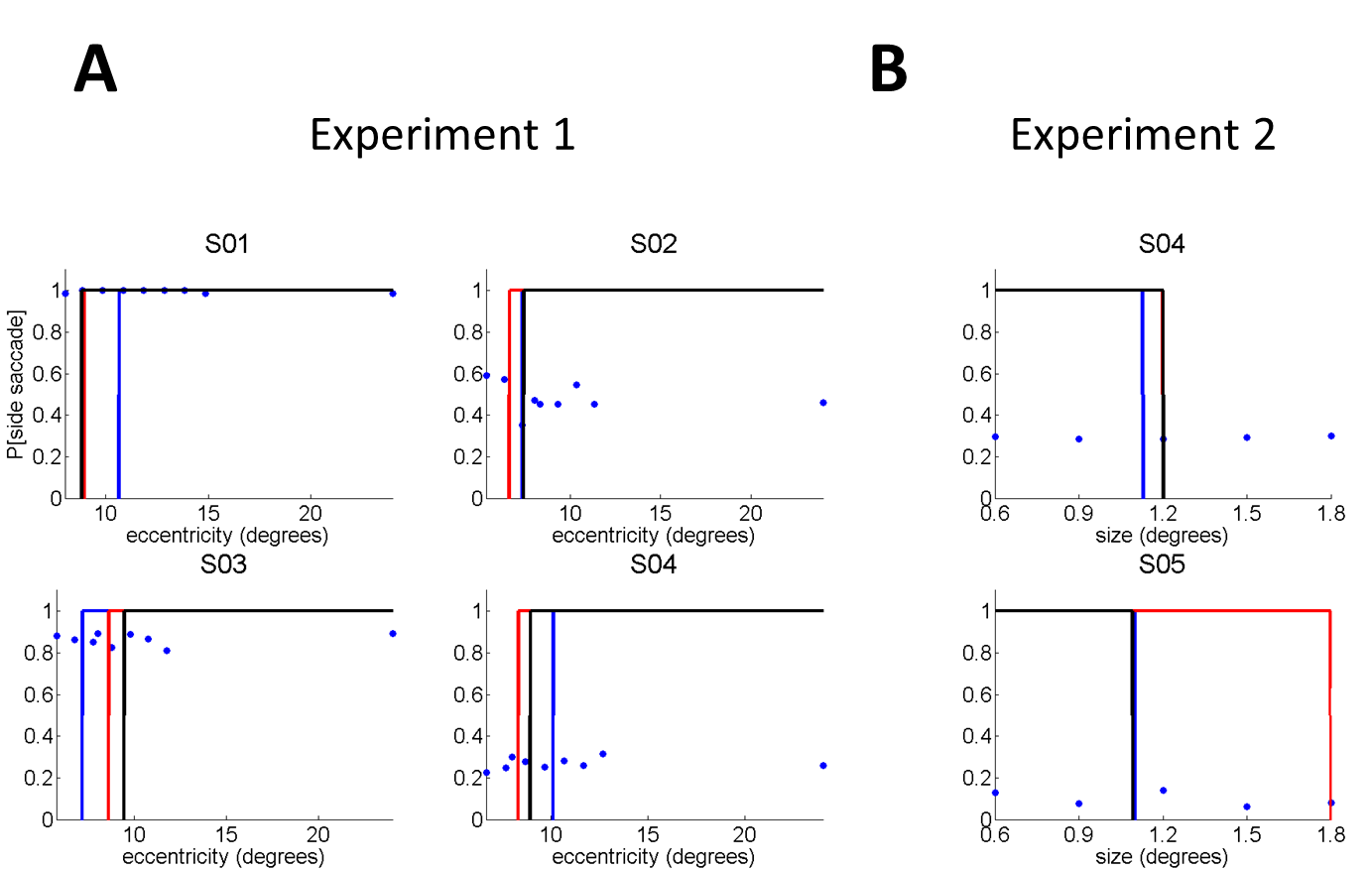
**

**Figure S3: Strategy choice.**
